# Metabolic Syndrome and Its Component Factors Among Corporate Company Employees in Kampala Uganda

**DOI:** 10.21203/rs.3.rs-4907042/v1

**Published:** 2024-09-11

**Authors:** Jemimah Kiboss Kyeyune, Zandile June-Rose Mchiza, Mwambi Bashir, Prossy Merab Ingabire, Florence Wamuyu Githinji, Florence Nakaggwa, Ivan Gabriel Busulwa, Rose Clarke Nanyonga

**Affiliations:** Clarke International University; South African Medical Research Council; Clarke International University; China-Uganda Friendship Hospital Naguru; Clarke International University; Clarke International University; IGB Health Consulting; Clarke International University

**Keywords:** Metabolic syndrome, corporate employees, principal components, factor analysis

## Abstract

**Background:**

Metabolic syndrome (MetS), a cluster of metabolic dysregulations indicative of increased cardiometabolic risk is on the rise in Sub-Saharan Africa. The study aimed to determine the prevalence of MetS and its components, among corporate employees in Kampala, Uganda.

**Methods:**

A cross-sectional survey was undertaken among 408 adults who were employees from seven corporate companies in Kampala, using the WHO STEPwise NCD screening approach. Metabolic syndrome was measured using the National Cholesterol Education Program Adult Treatment Panel (ATPIII) and the International Diabetes Federation (IDF) criteria with the waist circumference (WC) cut-off points adapted for Sub-Saharan African populations.

**Results:**

The mean population age (standard deviation [SD]) of the respondents was 34years (± 8.87) years and 52% of them were females. The prevalence of MetS was 22.8% (NCEP ATPIII) and 28.4% (IDF). Of the respondents who did not have MetS, 75% had at least one metabolic dysregulation. Of those respondents with MetS, only 31% perceived that they were at risk of this cluster of metabolic dysregulations. In this analysis, we observed that the systolic blood pressure (BP) and the body mass index (BMI) correlated strongly (r = 0.81 and r = 0.71) with the diastolic BP and waist circumference (WC), respectively. Age on the other hand correlated fairly with the WC and BMI (both r values = 0.46). Principal component analysis showed that the greatest loadings in principle factors one, two and three were from central obesity, with low HDL-C explaining 60.8% variance in the population. Age, BMI, family history of having cardiometabolic disorders, and perceived cardiometabolic disease risk (CMR) were associated with an increased risk of MetS by 5, 8.86, 1.55, and 2.73 (all P values were < 0.05) respectively in this group of respondents. These risks remained for age, BMI and perceived CMR after removing the confounding effects of education status, marital status and family history of cardiometabolic disease. Being single on the other hand, was associated with a reduced risk of MetS (0.23, p < 0.009).

**Conclusion:**

While the primary contributors to the high prevalence of MetS among corporate employees in Kampala, Uganda were the high BP, high WC and high fasting blood sugar (FBS); age, BMI and perceived CMR were the key determinants of MetS. Future MetS interventions should aim to control and monitor obesity indicators in this population. Additionally, the findings inform targeted screening parameters for cardiometabolic risk assessment and suggest the need for further research into a weighted algorithm for MetS in this population.

## BACKGROUND

Cardiometabolic diseases present with multiple conditions including diabetes mellitus, hypercholesterolemia, hypertension, and stroke (1). These diseases arise from complex interactions of risk factor clusters. Metabolic syndrome (MetS) is a cluster of metabolic dysregulations including insulin resistance, atherogenic dyslipidemia, central obesity, and hypertension that together indicate an increased risk of Type 2 diabetes and cardiovascular disease (1). Recent global burden of disease reports reveal that MetS components such as high blood pressure and high blood sugar (high fasting plasma glucose) were among the three leading risk factors for early death and poor health worldwide in 2021 (2).

Working adults spend most of their time in workplace environments where they are exposed to lifestyle related cardiometabolic disease risks. Their work conditions influence their self-regulatory capacity to make responsible decisions about personal diet and physical activity and potentially increasing their cardiometabolic risk (3). An example of such conditions is the observation from previous research that sedentary work increases risk of cardiovascular disease and death in apparently healthy working adults(4). The burden of MetS among working adults in Uganda is not well documented and possibly underestimated despite the cardiometabolic risk vulnerabilities present in this cohort. Current studies on MetS among adults in Uganda are from rural populations (5) except a few among a population of people living with HIV in Kampala (6, 7). To the best of our knowledge this is the first study of its kind to describe the prevalence and distribution of MetS and its determinants in a population of corporate company employees in Kampala, the capital city of Uganda.

## METHODS

### Study design

The study theoretical design (8) was etiognostic, seeking to investigate the occurrence of MetS as a function of past exposure to its determinants in the domain of corporate company employees. A cross-sectional survey was conducted involving multistage screening for MetS adapted from the WHO STEPWISE approach for surveillance of NCD risk factors (9). Employees of corporate companies aged 20 year – 55years formed the study target population.

### Study population

In 2021?, the estimated population of people employed by large (corporate) companies in Kampala was 83,969 as computed from the Uganda census of business establishments findings (10). The study sample size of 382 was determined by the Krejcie and Morgan formula for populations 75,000–1,000,000 (11). The final sample size adjusted for possible 10% non-response was 420.

Seven corporate companies that met the criteria of employing no less than 50 employees were purposively selected. Study participants were contacted through gatekeepers i.e., company executives who provided initial consent for inclusion of their companies in the survey. A quota sampling system was used for proportionate sampling of participants based on the staff size of the companies. Smaller companies had one screening day, larger companies had two or more screening days. Similarly, the size of the data collection team was adjusted depending on the approximate company quota and expected numbers of participants.

Company executives (gatekeepers) were briefed about the purpose and procedures of the study in a series of meetings. They were then provided with necessary information to share with the company staff. All employees within the companies were then sensitized about the study and invited to participate voluntarily through various company communication platforms such as email, social media and staff meetings. On survey days, company employees presenting at the screening locations between 8am and 11pm, who had fasted for at least 8 hours prior, were included in the study. Study participants who were pregnant or who had not fasted were excluded from the study. All study participants were provided with comprehensive information about the study, including potential risks and benefits. They were then requested to provide written consent prior to their participation, in accordance with the ethical principles outlined in the Declaration of Helsinki.

### Data collection

Data collection was conducted by the principal investigator and research assistants who received necessary training and detailed briefing on the purpose of the study as well as data collection procedures. A pilot study was conducted in a relatively small company that was not one of the 7 purposively selected companies included in the main study. The data collection team included professionally qualified clinical officers, a registered nurse, and laboratory technicians. These individuals had an understanding of the parameters under investigation due to their training. The data collection tool and procedures were adapted from the WHO STEP WISE approach for non-communicable disease surveillance manual - Step 3, and involved three broad measures: Questionnaires, objective physical and, biochemical measurements (9).

Study participants had their weight measured using a calibrated weighing scale to the nearest 0.5 kg with the participant not wearing shoes. The height was taken with the participant standing upright with the heel, buttock, and upper back along the same vertical plane against a measuring tape mounted on the wall (12, 13). Blood pressure was measured using a digital sphygmomanometer (Sinocare BSX516). Three consecutive readings of blood pressure were taken with the participant seated, each following a 5 min rest period. The average blood pressure reading was computed from the three readings(9).

Blood samples were collected from peripheral veins in participants’ arms by direct venipuncture. Fasting blood sugar was measured using the OnCall Plus digital glucometer. The blood samples were transported on the same day in cooler boxes to a registered biosafety level 2 laboratory in Kampala for the relevant lipid panel test using the cobas c 111 system.

The data was collected using a questionnaire which had sections for recording socio-demographic and other participant characteristics, and physical measurements and results from the biochemical tests. This tool was adapted from the WHO STEPS NCD risk surveillance instrument (9). The data collection forms from the lab indicated the study participant’s questionnaire code which also imprinted on the vacutainers so that the lab results were linked back to the original questionnaires.

### Data analysis

Data was entered into the online Kobo toolbox online database from where it was exported to Microsoft Excel for cleaning and then to R (version 4.3.3) in R studio for statistical analysis.

The study outcome was MetS determined using the NCEP ATP III and IDF algorithms (14, 15). These criteria have slightly varied cut off values for the 5 MetS components as outlined in Table 1 below. The NCEPTII has been adopted by previous studies in Uganda (7, 16) and Africa (17–19). However one study found that the IDF criteria was ideal for Ugandan populations because the regional waist circumference threshold was optimal for predicting MetS (20). In this study, we present the prevalence of MetS by both standards; however, data analysis was conducted with the data from the IDF MetS algorithm.

Additionally, previous research has shown greatest agreement between the NCEP ATPIII criteria and IDF in African populations (21). The Cohen’s kappa (k) was used to quantify the level of agreement between the NCEP ATPII and IDF criteria in this study. The Kappa result (22) was interpretated as follows: no agreement (values ≤ 0), none to slight (0.01 – 0.20), fair (0.21 – 0.40), moderate (0.41 – 0.60), substantial (0.61 – 0.80), and almost perfect agreement (0.81 – 1.00).

In addition to the MetS components mentioned above, obesity was assessed using the body mass index (BMI), calculated as weight in kilograms divided by height in meters squared (kg/m^2^). The BMI categories (24) were defined as follows: underweight (< 19 kg/m^2^), normal weight (19–24 kg/m^2^), overweight (25–29 kg/m^2^), and obese (> 30 kg/m^2^).

Exploratory analysis for descriptive statistics were conducted by obtaining relative and absolute frequencies and percentages for categorical variables. Measures of dispersion and central tendency were applied to continuous variables as follows: the arithmetic mean and standard deviations were used for normally distributed data, while the median and interquartile range (IQR) were used for skewed data. Data relating to age was tested for normality by the Shapiro-Wilk normality test (Shapiro’s value 0.963, p < 0.000). Some outliers however were observed in age with four of the respondents aged over 60 years old. Results relating to age are therefore presented using both mean and median where necessary to provide more information on age distribution and to increase comparability with results from similar studies.

The relationship between various risk factors and the study outcome (MetS) was examined through bivariate analysis. The correlations between the MetS components (central obesity, hypertriglyceridemia, low high-density lipoprotein cholesterol, high blood pressure and hyperglycemia) were assessed using Pearson correlation coefficients. The correlation coefficients were interpreted as follows (25): negligible correlation (r = 0.00 – 0.10), week correlation (r = 0.1 – 0.39), moderate correlation (r = 0.40 – 0.69), strong correlation ( r = 0.70 – 0.89) and very strong correlation (0.90 – 1.00). Additionally, association between participant intrinsic risk factors and MetS was analyzed by cross tabulations and chi-square statistical tests.

Multivariate analysis was conducted to identify the key risk factors for MetS as well as the principal MetS components. Principal component analysis was conducted and factors with the greatest contribution to each component were analyzed by observing components with the highest loadings. Subsequently these were rotated using Kaiser varimax rotation to identify components with loadings greater than one (thus having the highest contribution to each component). The percentage variance attributable to each factor was also assessed. Finally, sociodemographic and other intrinsic metabolic risk factors were included in logistic regression analysis to identify the key determinants of MetS in the study population.

All variables with a p-value less than 0.1 (26) in the bivariate analysis were further analyzed for possible correlation between risk factors. They were included in factors analysis and multiple regression models and relevant subgroup analyses. For each analysis, a 5% level of significance was utilized to determine the statistical significance of an independent variable. Consequently, each variable was deemed statistically significant if its associated p-value was less than 0.05, with a confidence interval of 95%.

## RESULTS

The response rate was 97.1% (n = 408) and none of the responses had completeness less than 10% hence there was no need to exclude observations. Average completeness was 96%. Further completeness of all the study variables included in the data analysis was checked and the maximum percentage of missing variables was 3.68 which was considered acceptable and expected to have negligible impact on the validity of the study findings (27, 28).

### Corporate company employees’ characteristics

The median age of study respondents was 34years (IQR 28–40) and nearly half 52% were females (Table 2). The average age for respondents with MetS was 40.9years (SD 9.2) and for respondents without MetS it was 33.6years (SD 8.07). Nearly half the respondents were either married or cohabiting 56% (n = 225) and 41% (n = 163) were single. Most of the respondents had tertiary level education 65% (n = 254), with 46% (n = 183) being departmental staff and most of the others either managers 23% (n = 92) or support staff 28% (n = 112). A small number, 2% (n = 24), were stationed at the company by security personnel. Half the respondents knew of close family members who had a cardiometabolic disease. Only 18% (n = 72) of the respondents perceived themselves to be at risk of getting a cardiometabolic disease while 14% (n = 58%) of respondents had a known cardiometabolic disease. Most of the respondents (x%) were overweight (n = 240) while the mean BMI in the study population was 26kg/m^2^. The details of the individual characteristics of the study population are outlined in Table 2.

### Prevalence of Metabolic syndrome

The prevalence of MetS was 22.8% (n = 93) by the NCEP ATPIII definition and 28.4% (n = 116) by the IDF criteria. The Cohen Kappa value of 0.85 showed strong agreement between the two criteria (p < 0.000).

### Bivariate analysis

The findings show a strong association (p < 0.001) between MetS and Age as well as BMI (Additional file 1). A higher proportion of participants with MetS were female 60.2% (n = 56), however this difference was not statistically significant (p = 0.1).

Over three quarter of the participants with MetS were aged above 35years (77.2%, n = 71). Most participants over 35 years old (82.4%, n = 150) had BMI over 25kg/m^2^ and overall, 67% (n = 271 of the population had overweight BMI. Among those with an overweight BMI, only 21.1% (n = 56) perceived themselves to be at risk of cardiometabolic illness, while 85.1% (n = 97) of those with MetS had overweight BMI. Marital status was significantly associated with MetS, with 35% (n = 79) of married/cohabiting participants having MetS compared to 18% (n = 30) of single participants (p = 0.002). Age was also significantly associated with MetS (P < 0.001). There was no statistically significant difference in the prevalence of MetS across different employee levels (p = 0.6). However, 79% (n = 73) of managers had overweight BMI and 33.7% (n = 31) had MetS compared to 54% (n = 61) and 26%% (n = 29) for support staff respectively. Notably, only 44.6% (n = 50) of support staff were aged over 35years compared to 69.6% (n = 64) of managers.

### Prevalence of MetS components according to the IDF criteria

Most of the participants 81.9% (n = 334) had at least one metabolic dysregulation and the greatest proportion 53.4% (n = 218) had 1–2 components compared to those who had more than 3 28% (n-116). The most prevalent MetS component (Additional file 2) by IDF criteria was high fasting blood sugar 82.8% (n = 77). Fourteen percent (n = 13) of the participants had blood sugar levels indicative of diabetes (> 7.1 mmol/L). Of the participants with cardiometabolic disease, 63.6% of those with diabetes and 59.5% of those with hypertension were not aware of their respective conditions. The second most prevalent MetS component was high waist circumference affecting 75.3% (n = 70) of participants, followed by hypercholesterolemia with about 60% having high triglycerides (n = 55) and a similar proportion having low HDL-C (n = 57). One-third of the participants had hypertension (n = 29). Both high waist circumference and hypertension were significantly associated with having an overweight BMI (p < 0.001) and age (p = 0.001).

### Correlations between the MetS components

Systolic and diastolic blood pressure showed the highest correlation (r = 0.81). The correlation between triglycerides and fasting blood sugar, as well as waist circumference, and between systolic blood pressure and waist circumference, were moderately high (r = 0.39). The other metabolic components had weaker correlations below 0.4 (Table 3). Most of the correlations were statistically significant with p < 0.05. BMI had the highest correlations with fasting blood sugar levels and waist circumference (r = 0.71). Age had the highest correlations with both fasting blood sugar levels and waist circumference (r = 0.46).

**Figure 2:**
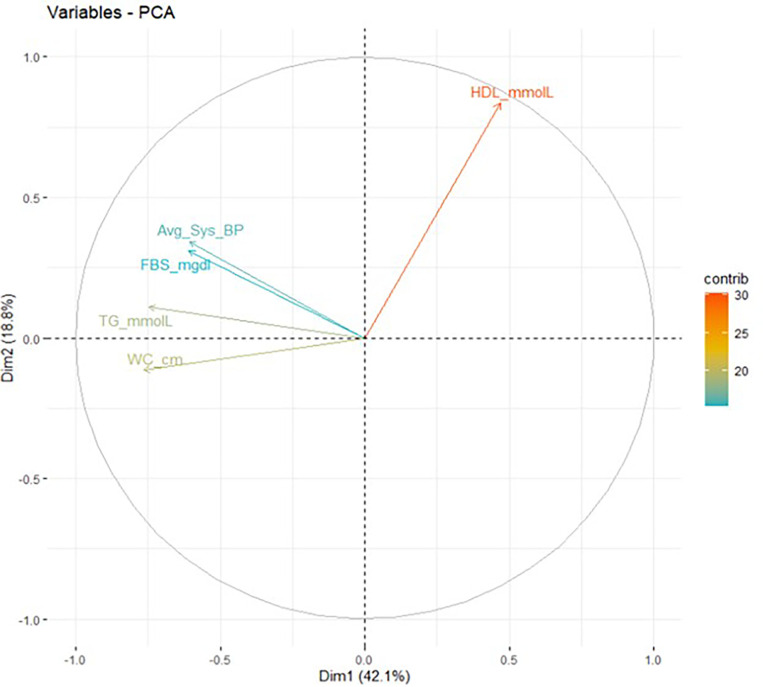
Principal component analysis model showing components that explain variance in the study population (Abbreviations: SysBP – systolic blood pressure, TG – Triglycerides, HDL – High density lipoprotein cholesterol, FBS – Fasting blood sugar, WC – Waist circumference.)

In a six-factor principal component analysis model (Fig. 2 below), the most significant contributors on the first and second dimensions were: hyperglycemia, systolic BP, triglyceride levels, and WC explaining 60.9% of the variation observed.

### MetS principal component analysis (PCA)

Exploratory factor analysis for MetS factors in the study population based on eigen values and a scree-plot (not shown) revealed that two factors explained 60.8% of the variance in the population. Principal component analysis further showed that the greatest loadings in principal factor one, two and three were from central obesity, low HDL-C and high average systolic BP respectively as shown in Table 4 below.

#### Factors with highest loadings are bolded.

Kaiser-varimax rotation (not shown here) revealed that hyperglycemia, HDL-C and systolic blood pressurehad factor loadings greater than one in components one, two and three respectively.

### Logistic regression analysis

The logistic regression analysis (Table 5) reveals that age, BMI, family history, and perceived CMR increased the risk of corporate employees to present with MetS by 5, 8.86, 1.55, and 2.73 respectively (all p < 0.05). Being single on the other hand reduced the likelihood by 0.23 (p < 0.009).

Table 5 Logistic regression analysis showing risk factors associated with MetS

## DISCUSSION

This study sought to determine the prevalence of MetS in a population of corporate company employees in Kampala Uganda. Additionally, the researchers evaluated the prevalence and possible relationships between MetS components as well their contribution of MetS components to occurrence of MetS. The findings of the study are discussed in relation to previous research with similar focus in comparable settings.

The 28.4% prevalence of MetS (IDF) in this study was higher than the previously estimated 18% prevalence of MetS (IDF) for the general population in sub-Saharan Africa (29). The prevalence 22.8% by NCEP ATP III was higher than the 19.2% (ATP III) prevalence from previous research in a rural population in Uganda(5). However, this prevalence is comparable to similar studies in sub-Saharan Africa (SSA), e.g., among working adults in Nigeria 24.2% (30) and Ethiopia 27.6% (31). These findings indicate a high prevalence of MetS in this population, comparable to similar populations elsewhere in sub–Saharan Africa, suggesting greater vulnerability among working adults possibly due to occupation or urbanicity, or both.

In addition to the high prevalence of the clustering of the CMDs MetS, the findings also reveal a high prevalence of individual cardiometabolic disorders in this population. For instance, while previous Uganda evidence (32) has shown the prevalence of diabetes mellitus in the general population to be low, at 1.4%, 14% of participants in the current study had fasting blood sugar readings higher than 7.0mmol/L. Nearly a third (29%) of the study participants had blood pressure readings indicative of hypertension, slightly higher than the nationwide observation of 26.4% from a previous survey in Uganda (33). The results thus emphasize the urgent need for interventions to mitigate cardiometabolic disease risk in this population.

Cardiometabolic risk perception in the population was low with only 31.9% of participants with MetS and only 21.1% of those who were overweight perceiving themselves to be at risk of cardiometabolic disease. Additionally, most of the participants with diabetes and hypertension were not aware of their conditions. Previous studies in Uganda similarly found very low awareness levels among participants with hypertension (33) and impaired fasting glucose (32). These findings highlight a low cardiometabolic risk perception among a population otherwise at high risk of cardiometabolic disease.

Despite the apparent low awareness, cardiometabolic risk perception was strongly associated with MetS and presented higher odds of MetS (OR 1.92, CI 1.00–3.69). This strong association remained even when controlled for education status, age and family history of cardiometabolic disease. This suggests that risk perception alone was not protective against MetS in this population. Previous studies found that knowledge and awareness of MetS alone do not result in behavior change (34), so indicating a need for further research to understand the possible environmental factors and other antecedents that contribute to MetS risk in this setting.

There was a high prevalence of metabolic dysregulations with 81.9% of the participants having at least one metabolic issue, and most of the participants (53.4%) having one or two. This finding suggests an opportunity for successful MetS prevention interventions, as although most of the participants are at high risk, the majority do not yet have the condition. The main contributors to MetS identified in the factor analysis principal component one included waist circumference and fasting blood sugar, which were also significantly correlated. Additionally main contributions to variance in the population were noted from systolic blood pressure, similar to findings from a previous study in Iran (35). These findings align with previous studies in SSA which found that high blood pressure and waist circumference were key components (36 – 38) of MetS, and a previous study in Uganda (32) which found that impaired fasting glucose was associated with BMI.

Further observation from the principal component analysis (PCA) revealed that low HDL-C was also a key contributor to principal component two explaining 18.8% variance in the population. This differs from a previous MetS factor analysis study (35) study that showed high contributions of both low HDL-C and high triglycerides to the same principal component. The findings however demonstrate an independent contribution of low HDL-C as risk factor for MetS in this study population thus supporting a conclusion from a previous multiethnic study (39).

Metabolic syndrome in this study was found to be strongly associated with age above 34 years and BMI above 25kg/m^2^, similar to previous findings in Uganda regarding BMI, and to previous studies among working adults in Nigeria (38) regarding age. However, it is notable that previous studies among working adults in Africa reported higher mean ages, typically above 40years, compared to the average age of 34years observed in this study. A previous study (19) among working adults observed a higher mean age of 48.74 (SD 8.25) for participants with MetS, whereas the mean age in this study was 40.9years (SD 9.2). This reflects the younger population in Uganda compared to other countries (40) and highlights the need for early intervention in the young adult age group 20–39years age group (41) to mitigate MetS in this population.

Having overweight BMI presented significantly high odds 8.35 (CI 3.75–21.54) in a population where the average BMI was 26kg/m^2^, with 67% of all participants and 80.8% of those aged over 34years having overweight BMI. The very high odds of MetS in overweight participants remained even when adjustment was made for age, family history, marital status education level. This prevalence is considerably higher than the preliminary findings of the most recent demographic and health survey in Uganda (42), which reported 26.4% of adult women and 10.7% of adult men being overweight. These findings indicate a very high prevalence of overweight BMI that is strongly associated with MetS in this population.

## CONCLUSION

The findings suggest a higher prevalence of MetS in working adults, accompanied by a low perceived risk of cardiometabolic disease, which may result in poor uptake of much needed protective lifestyle changes. Although the prevalence of MetS was high and a majority of respondents had at least one metabolic dysregulation, most had only one or two MetS components thus presenting an opportunity for effective prevention interventions. The relatively young age of the working population observed in this study and the occurrence of MetS at a comparatively young age underscores the need for early interventions to mitigate cardiometabolic disease risk in this group. Considering that the main components and determinants of MetS include blood pressure, waist circumference, age and BMI; incorporating these factors into a rapid office-based screening algorithm could facilitate greater awareness and monitoring of MetS risk in this population. Further research is recommended to explore the possible lifestyle and environmental factors contributing to the high prevalence of MetS in this population. Finally, the findings suggest a need for development of a weighted algorithm for MetS as its components have differing contributions to variance in the population.

## Figures and Tables

**Figure 1:**
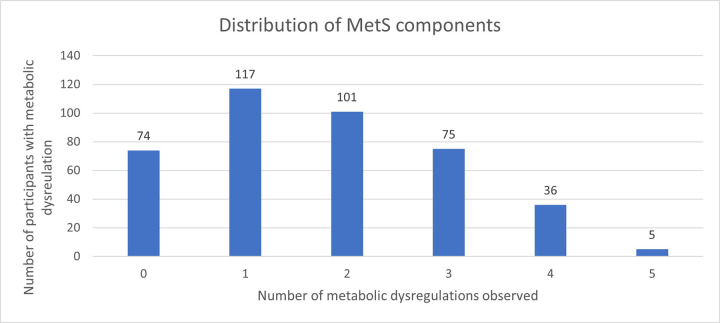
Distribution of the numbers of Metabolic components observed in the study population

**Table 1:** Metabolic syndrome components standard cut off thresholds

	NCEP ATP III	IDF
Prerequisite	None	WC ≥ 94 cm (men), WC ≥ 80 cm (women)†
No of criteria	≥ 3 of	and ≥ 2 of
Obesity	WC ≥ 102 cm (men), WC ≥ 88 cm (women)	
BP (mmHg)	≥ 130/85‡	≥ 130/85‡
HDL-C (mmol/l)	< 1.0 (men), < 1.3 (women)‡	< 1.0 (men), < 1.3 (women)‡
TG (mmol/l)	≥ 1.7‡	> 1.7‡
Glucose (mmol/l)	≥ 6.1‡	≥ 5.6‡

Abbreviations: Blood pressure - BP, High density lipoprotein cholesterol - HDL-C, Triglycerides- TG, or treatment for this abnormality - ‡.

IDF recommended waist circumference threshold for Sub-Saharan Arican populations (23) - †

**Table 2:** Descriptive statistics: simple frequencies of study population characteristics

Characteristic	N=408^1^
	**Median (IQR)**
Age	34 (28, 40)
	**n (%)**
Sex	
Female	213 (52%)
Male	195 (48%)
Home Region	
Central	49 (12%)
Eastern	108 (27%)
Northern	111 (28%)
Western	124 (31%)
Other	8 (2.0%)
Marital Status	
Divorced/Separated	14 (3.5%)
Married/Cohabiting	225 (56%)
Single	163 (41%)
Education level	
Primary	34 (8.7%)
Secondary	81 (21%)
Tertiary	254 (65%)
Staff level	
Departmental staff	183 (46%)
Manager	92 (23%)
Other	12 (3.0%)
Support staff	112 (28%)
Family History of CMD	200 (50%)
Any known CMD	58 (14%)
Perceived Cardiometabolic risk	72 (18%)
BMI over 25kg/m^2^	240 (60%)
	**Median (IQR)**
BMI	26.1 (22.9, 29.4)

(NOTE: This table was longer than an A4 page hence presented at the end of the paper but should be inserted in the in results section just after the subsection “Corporate company employees characteristics”)

**Table 3:** Correlations showing relationship between MetS components based on IDF algorithm

	SBP (mmHG)	DBP (mmHG)	TG (mmol/L)	HDL-C (mmol/L)	FBS (mmol/L)	WC (cm)	BMI (kg/m2)	Age (years)
**SBP (mmHG)**	1							
**DBP (mmHG)**	0.81	1						
**(p-value)**	0.000							
**TG (mmol/L)**	0.29	0.31	1					
**(p-value)**	0.000	0.000						
**HDL-C_mmol/L**	−0.08	−0.03	−0.22	1				
**(p-value)**	0.134	0.543	0.000					
**FBS (mmol/L)**	0.19	0.19	0.39	−0.11	1			
**(p-value)**	0.000	0.000	0.000	0.027				
**WC (cm)**	0.39	0.3	0.39	−0.3	0.28	1		
**(p-value)**	0.000	0.000	0.000	0.000	0.000			
**BMI (kg/m2)**	0.32	0.26	0.25	−0.22	0.71	0.71	1	
**(p-value)**	0.000	0.000	0.000	0.000	0.000	0.000		
**Age (yrs)**	0.28	0.28	0.23	−0.15	0.46	0.46	0.31	1
**(p-value)**	0.000	0.000	0.000	0.003	0.012	0.000	0.000	

**Abbreviations:** SBP - systolic blood pressure, DBP - Diastolic blood pressure, TG - Triglycerides, HDL-C - High density lipoprotein cholesterol, FBS - Fasting blood sugar, WC - Waist circumference, BMI - Body mass index.

**Table 4:** Principal Component Analysis showing principal MetS components' factor loadings

	PCA					
	Initial loadings			Contributions from squared loadings		
Variable	PC1	PC2	PC3	PC1	PC2	PC3
**Average systolic BP**	−0.419	0.355	**0.658**	0.175	0.126	**0.433**
**Triglycerides**	−0.514	0.113	−0.262	0.264	0.013	0.069
**High density lipoprotein**	0.323	**0.863**	0.042	0.104	**0.745**	0.002
**Fasting Blood sugar**	−0.421	0.32	−0.648	0.177	0.102	0.421
**Waist circumference**	**−0.528**	−0.118	0.276	**0.279**	0.014	0.076
**Eigen values**	2.104	0.938	0.856			
**Percentage variance**	42.1	18.8	17.1			
**Cumulative variance**	42.1	60.8	78			

Factors with highest loadings are bolded.

## Data Availability

The datasets during and/or analyzed during the current study are available from the corresponding author on reasonable request.
